# Fasting plasma glucose in the first trimester is related to gestational diabetes mellitus and adverse pregnancy outcomes

**DOI:** 10.1007/s12020-021-02831-w

**Published:** 2021-08-03

**Authors:** Jia-Ning Tong, Lin-Lin Wu, Yi-Xuan Chen, Xiao-Nian Guan, Fu-Ying Tian, Hua-Fan Zhang, Kan Liu, Ai-Qi Yin, Xiao-Xia Wu, Jian-Min Niu Prof

**Affiliations:** Department of Obstetrics, Shenzhen Maternity & Child Healthcare Hospital, The First School of Clinical Medicine, Southern Medical University, Hongli Road, Futian District, Shenzhen, Guangdong Province China

**Keywords:** The First Trimester FPG, GDM, Metabolic Diseases in Pregnancy, Adverse Pregnancy Outcomes

## Abstract

**Purpose:**

To investigate and identify first-trimester fasting plasma glucose (FPG) is related to gestational diabetes mellitus (GDM) and other adverse pregnancy outcomes in Shenzhen population.

**Methods:**

We used data of 48,444 pregnant women that had been retrospectively collected between 2017 and 2019. Logistic regression analysis was used to evaluated the associations between first-trimester FPG and GDM and adverse pregnancy outcomes, and used to construct a nomogram model for predicting the risk of GDM. The performance of the nomogram was evaluated by using ROC and calibration curves. Decision curve analysis (DCA) was used to determine the clinical usefulness of the first-trimester FPG by quantifying the net benefits at different threshold probabilities.

**Results:**

The mean first-trimester FPG was 4.62 ± 0.42 mmol/L. A total of 6998 (14.4%) pregnancies developed GDM.489(1.01%) pregnancies developed polyhydramnios, the prevalence rates of gestational hypertensive disorder (GHD), cesarean section, primary cesarean section, preterm delivery before 37 weeks (PD) and dystocia was 1130 (2.33%), 20,426 (42.16%), 7237 (14.94%), 2386 (4.93%), and 1865 (3.85%), respectively. 4233 (8.74%) of the newborns were LGA, and the number of macrosomia was 2272 (4.69%), LBW was 1701 (3.51%) and 5084 (10.49%) newborns had admission to the ICU, which all showed significances between GDM and non-GDM groups (all *P* < 0.05). The univariate analysis showed that first-trimester FPG was strongly associated with risks of outcomes including GDM, cesarean section, macrosomia, GHD, primary cesarean section, and LGA (all OR > 1, all *P* < 0.05), furthermore, the risks of GDM, primary cesarean section, and LGA was increasing with first-trimester FPG as early as it was at 4.19–4.63 mmol/L. The multivariable analysis showed that the risks of GDM (ORs for FPG 4.19–4.63, 4.63–5.11 and 5.11–7.0 mmol/L were 1.137, 1.592, and 4.031, respectively, all *P* < 0.05) increased as early as first-trimester FPG was at 4.19–4.63 mmol/L, and first-trimester FPG which was also associated with the risks of cesarean section, macrosomia and LGA (OR for FPG 5.11–7.0 mmol/L of cesarean section: 1.128; OR for FPG 5.11–7.0 mmol/L of macrosomia: 1.561; OR for FPG 4.63–5.11 and 5.11–7.0 mmol/L of LGA: 1.149 and 1.426, respectively, all *P* < 0.05) and with its increasing, the risks of LGA increased. Furthermore, the nomogram had a C-indices 0.771(95% CI: 0.763~0.779) and 0.770(95% CI:0.758~0.781) in training and testing validation respectively, which showed an acceptable consistency between the observed, validation and nomogram-predicted probabilities, the DAC curve analysis indicated that the nomogram had important clinical application value for GDM risk prediction.

**Conclusions:**

FPG in the first trimester was an independent risk factor for GDM which can be used as a screening test for identifying pregnancies at risk of GDM and adverse pregnancy outcomes.

## Introduction

Gestational diabetes mellitus (GDM) refers to an abnormality of glycometabolism that occurs for the first time in the second or third trimester of pregnancy and does not include type 1 or type 2 diabetes, which exists before pregnancy [1]. GDM is associated with adverse maternal and fetal outcomes and maternal complications in pregnancy and later in life. The prevalence of GDM is increasing; this is closely linked to the prevalence of obesity and type 2 diabetes in specific countries, and the prevalence of obesity among women of childbearing age partly explains this increase. The risks posed to mothers with GDM range from direct pregnancy complications, particularly the need for cesarean section and risk of gestational hypertension, to their lifetime risk of developing type 2 diabetes and cardiovascular diseases. Regarding their children, there is an increased short-term risk of obesity, premature birth, shoulder dystocia, and neonatal hypoglycemia, as well as a long-term risk of obesity and abnormal plasma glucose (PG) metabolism. Therefore, GDM is associated with a particularly poor prognosis [1–3] and early detection of GDM is of great importance to help with prevention and treatment.

Epidemiological studies of hyperglycemia and adverse pregnancy outcomes (HAPO) in multiple countries have recommended that a fasting plasma glucose (FPG) value of 5.1 mmol/L (92 mg/dL) in the first trimester can be the threshold for elevated blood glucose. It also indicated that if FPG ≤ 4.4 mmol/L (80 mg/dL), the risks of some adverse pregnancy outcomes are low [4]. Furthermore, several researchers examined whether first-trimester FPG is also consistently associated with obstetric complications, and a retrospective study of 6129 pregnant women by Riskin–Masiah who observed first-trimester FPG found that FPG is associated with adverse outcomes and risk of GDM [5]. Therefore, it is valuable to provide more data about first-trimester FPG from a single medical database where there might be some homogeneity in the patient population. Due to metabolic changes during pregnancy, blood glucose between 6 and 10 weeks in the first trimester can drop by ∼2 mg/dL, and many scholars have pointed out that a specific lower limit of first-trimester FPG should be defined [6].

This study hoped to provide new evidence which could identify the relationships with first-trimester FPG, GDM, and other obstetrical outcomes in the Shenzhen population.

## Materials and methods

**Fig. 1:**
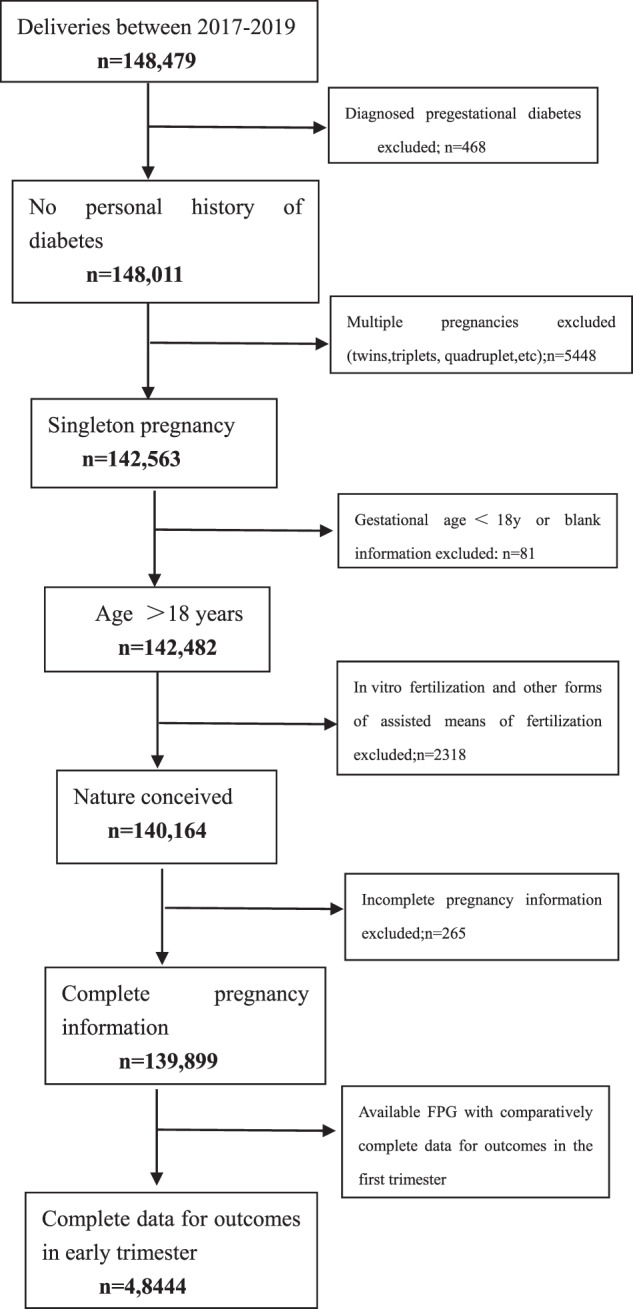
Flow chart of selecting process of the survey

This survey was an analysis of retrospectively collected data from the clinical database of the Shenzhen Maternity and Child Healthcare System between 2017 and 2019. Patients younger than 18 years old or with incomplete information, diagnosed pregestational diabetes, multiple pregnancies, or pregnancies conceived by assisted reproductive technology were excluded. Patients included were singleton pregnancies who attended our hospital to establish a maternal-fetal manual in the first trimester, performed regular visits and gave birth in our hospital. They also received routine FPG testing in the first trimester. Finally, the selected patients included only those with an available FPG in the first trimester (<14 weeks) performed under the standard conditions and who had complete data on all outcomes (Fig. 1). All patients were managed according to standard clinical protocols, and throughout the research periods, protocols were in accordance with the screening and management of GDM, followed by the recommendation of the International Diabetes and Pregnancy Research Group (IADASG) [1]. According to a previous study, the incidence of GDM in pregnant women was about 10~15%, with an OR of 3.3 [7], with this assumption, 22,780 pregnant women would yield 80% power to show an incidence of GDM of 15%, considering a dropout rate 20%, a total sample size required for the study was 27,336.

### Diagnostic criterion

#### Gestational diabetes mellitus (GDM)

American Diabetes Association (ADA) has been using the one-step approach of the IADASG as the screening and diagnostic standard for GDM since 2011; here, in this study, the 2019 reviewed version was used [8]. Specifically, the IADPSG recommends that all pregnant women with no previous history of diabetes take a 75-g oral glucose tolerance test (OGTT) at 24 to 28 gestational weeks. Any value above baseline before glucose consumption (0 h) or PG levels at 1 h and 2 h after glucose consumption that are abnormal were diagnosed as GDM, namely, 0 h ≥ 5.1 mmol/L (92 mg/dl), 1 h ≥ 10.0 mmol/L (180 mg/dl), and 2 h ≥ 8.5 mmol/L (153 mg/dl).

#### Gestational hypertensive disorder (GHD)

Preeclampsia was defined as systolic pressure ≥140 mm Hg or diastolic pressure ≥90 mm Hg on two or more occasions a minimum of 6 h apart, proteinuria ≥1+ or more on a dipstick test, or urine protein ≥300 mg for a 24-h period. Gestational hypertension was diagnosed when elevated blood pressure met the criteria but without protein urine [9].

#### Prepregnancy body mass index (BMI)

To calculate BMI, prepregnancy weight (kg) was divided by the squared height (m^2^). Prepregnancy BMI was categorized according to the WHO standard [10]: women were underweight (BMI < 18.5 kg/m^2^), normal (18.5–25 kg/m^2^), overweight (25–30 kg/m^2^), obese ≥30 kg/m^2^), obese grade 1 (30–35 kg/m^2^), obese grade 2 (35–40 kg/m^2^), or obese grade 3 (≥40 kg/m^2^).

#### Gestational weight gain

The gestational weight gain (GWG) in kg of the first trimester was calculated as the weight at 13^+6^ gestational weeks minus the prepregnancy weight. The GWG of the first trimester was categorized by the IOM (Institute of Medicine) standard [11]: inadequate (GWG < 0.5 kg), adequate (GWG 0.5–2.0 kg), and excessive (GWGå 2.0 kg).

#### Macrosomia, large for gestational age (LGA) and low birth weight (LBW)

Macrosomia was defined as a newborn weight in g ≥ 4000. Large for gestational age (LGA) was defined as newborn birth weight of above the 90th percentile if the birth weight was greater than the estimated 90th percentile for the same gestational age. Low birth weight (LBW) was defined as newborn birth weight <2500 g [4, 12].

All patients were considered when analyzing GDM and FPG in the first trimester. To analyze other obstetrical and maternal-fetal outcomes, patients with GDM were excluded to avoid bias arising from different treatments for GDM.

### Data collection

We collected the descriptive statistics, clinical biochemical information, and pregnancy outcomes of the patients. The descriptive statistics referred to age, height prepregnancy BMI, etc. Pregnant women generally had their first visits at gestational weeks 9–13^+6^. Clinical and biochemical data were collected retrospectively from the first prenatal visit, and data about the neonatal outcomes were collected after birth and saved into standardized maternal-natal information systems for the following statistical analysis. Clinical information also covered a history of hypertension and diabetes, among other conditions. Pregnancy outcomes included complications for pregnancies and newborns.

### Diagnostic method

The OGTT and FPG results were measured by using the enzyme electrode method (DXC800, Beckman). The standard laboratory procedure is to centrifuge samples within 20 min of collection. The results were collected retrospectively from the report system of the laboratory.

### Outcomes

The obstetrical adverse outcomes included GDM, cesarean section, primary cesarean section, polyhydramnios, preterm delivery before 37weeks (PD), dystocia, and GHD, which included high blood pressure during pregnancy and preeclampsia. The neonatal outcomes included macrosomia, LGA, LBW, and ICU attendance of newborns. The main outcomes for this survey were the risk of GDM, primary cesarean section, and LGA, while the others were secondary outcomes.

### Statistical analysis

Analyses were performed using *R* statistical software version 3.6.1. Continuous variables were presented as the means with standard deviations, while categorical data were expressed as counts and percentages. Summary statistics between both groups were compared using either unpaired Student’s *t* test or Mann–Whitney tests for continuous data, and chi-squared tests or Fisher’s Exact test for categorical data. Univariate and multivariable-adjusted odds ratios (OR) with 95% confidence interval (CI) of FPG for associations between first-trimester FPG and GDM and adverse pregnancy outcomes were estimated using the logistical regression model. Nomogram and calibration curve were performed with the “rms” package, then, a nomogram diagram for predicting the risk of GDM with first-trimester FPG for GDM was established by using the stepAIC filter variables, which nomogram model was used for predicting the risk of GDM and enabling the user to easily compute output probabilities. Decision curve analysis (DCA) was performed with the “dca” package, which was conducted to determine the clinical usefulness of the first-trimester FPG nomogram by quantifying the net benefits at different threshold probabilities. A *p* value of < 0.05 was considered to indicate statistical significance.

## Results

### Baseline demographic and adverse outcome

The baseline demographic and adverse outcomes according to the presence of GDM were summarized in Table 1. Among 148,479 pregnancies delivered between 2017 and 2019, a total of 48,444 pregnant women were included in this study and 6,998(14.4%) pregnancies were diagnosed as GDM. The mean maternal age was 30.85 ± 4.04 years, which showed a significant difference between non-GDM and GDM groups (30.57 ± 3.94 vs. 32.52 ± 4.22, *P* < 0.001). The pregestational BMI was 20.65 ± 2.65 kg/m2, which was higher in GDM groups (20.50 ± 2.56 vs. 21.69 ± 3.01, *P* < 0.001) when compared with non-GDM groups. And first-trimester FPG was 4.62 ± 0.42 mmol/L, of which 12.18% were first-trimester FPG ≤ 4.19 mmol/L, 73.29% were 4.19–4.62 mmol/L,37.61% were 4.63–5.10 mmol/L, and 9.31% were 5.11–7.0 mmol/L, and the results indicated that first-trimester FPG was higher in GDM groups(*P* < 0.001). For newborns, the weight of newborns was 3281.52 ± 440.43 g, which was a significant difference between non-GDM and GDM groups (3284.19 ± 434.95 vs. 3265.74 ± 471.30, P < 0.001), the scores of Apgar 1 min and Apgar 5 min was 9.92 ± 0.50 and 9.99 ± 0.63, respectively. Four hundred and eighty-nine (1.01%) pregnancies developed polyhydramnios, the prevalence rates of GHD, cesarean section, primary cesarean section, PD, and dystocia was 1130 (2.33%), 20426 (42.16%), 7237 (14.94%), 2386 (4.93%), and 1865 (3.85%), respectively, which all showed significances between two groups (all *P* < 0.05). Four thousand two hundred and thirty-three (8.74%) of the newborns were LGA, and the number of macrosomia was 2272 (4.69%), LBW was 1701 (3.51%), and 5084 (10.49%) newborns had admission to the ICU, all the prevalence rates were higher in the GDM group than those in the non-GDM groups (all *P* < 0.05).

**Table 1:** Baseline demographic and adverse pregnancy outcome

Characteristics	Overall	No GDM	GDM	*P* value
*n*	48,444	41,446 (85.6%)	6998 (14.4%)	
Maternal Characteristics				
Maternal age, years^a^	30.85 ± 4.04	30.57 ± 3.94	32.52 ± 4.22	<0.001
Height, m	1.60 ± 0.07	1.60 ± 0.07	1.59 ± 0.07	<0.001
Prepregnancy BMI, kg/m^2^	20.65 ± 2.65	20.50 ± 2.56	21.69 ± 3.01	<0.001
Category of prepregnancy BMI ^b^(*n*, %)				<0.001
≤18.5 kg/m^2^	7530 (20.5)	6960 (21.6)	570 (12.4)	
18.5–24.9 kg/m^2^	26,941 (73.3)	23,513 (73.1)	3428 (74.5)	
25.0–29.9 kg/m^2^	2099 (5.7)	1552 (4.8)	47 (11.9)	
30.0–34.9 kg/m^2^	163 (0.4)	116 (0.4)	47 (1.0)	
35.0–39.9 kg/m^2^	17 (0.0)	12 (0.0)	5 (0.1)	
≥40.0 kg/m^2^	7 (0.0)	5 (0.0)	2 (0.0)	
FPG in first trimester, mmol/L	4.62 ± 0.42	4.59 ± 0.39	4.80 ± 0.55	<0.001
Category of FPG in first trimester (n, %)				<0.001
≤ 4.19 mmol/L	5889 (12.2)]	5355 (12.9)	534 (7.7)	
4.19–4.62 mmol/L	19,770 (40.9)	17,621 (42.6)	2149 (30.9)	
4.63–5.10 mmol/L	18,184 (37.6)	15384 (37.2)	2800 (40.2)	
5.11–7.0 mmol/L	4503 (9.3)	3029 (7.3)	1474 (21.2)	
Delivery times	0.37 ± 0.53	0.35 ± 0.51	0.49 ± 0.57	<0.001
OGTT at 0 h, mmol/L^d^	2.52 ± 2.17	2.42 ± 2.14	3.10 ± 2.23	<0.001
OGTT at 1 h, mmol/L^d^	4.41 ± 3.87	4.08 ± 3.66	6.35 ± 4.51	<0.001
OGTT at 2 h, mmol/L^d^	3.91 ± 3.41	3.62 ± 3.22	5.61 ± 3.99	<0.001
Delivery mode (n, %)				<0.001
Obstetric forceps	282 (0.6)	234 (0.6)	48 (0.7)	
Eutocia	27,294 (56.3)	23,799 (57.4)	3495 (49.9)	
Vacuum extraction	424 (0.9)	347 (0.8)	77 (1.1)	
Breech presentation	18 (0.0)	18 (0.0)	0 (0.0)	
Cesarean section	20,426 (42.2)	17048 (41.1)	3378 (48.3)	<0.001
**Primary cesarean section**	**7237 (14.9)**	**5691 (13.7)**	**1546 (22.1)**	**<0.001**
Gestational weight gain (GWG), Kg	2.21 ± 2.58	2.21 ± 2.57	2.20 ± 2.60	0.768
Category of GWG^c^ (n, %)				0.264
<0.5 kg	6212 (22.8)	5280 (22.7)	932 (23.5)	
0.5–2 kg	7798 (28.6)	6703 (28.8)	1095 (27.7)	
0.5–2 kg	13227 (48.6)	11296 (48.5)	1931 (48.8)	
Major birth malformation of past history (*n*, %)	9062 (18.7)	7509 (18.1)	1553 (22.2)	<0.001
Bleeding amount in 24 h, mL	287.82 ± 98.80	287.68 ± 97.77	88.64 ± 104.72	0.455
Newborn characteristics				
Gestational age at delivery, wk	38.88 ± 1.48	38.93 ± 1.47	38.58 ± 1.48	<0.001
Weigh of newborn, g	281.52 ± 440.43)	284.19 ± 434.95	3265.74 ± 471.30	0.001
Apgar 1 min	9.92 ± 0.50	9.92 ± 0.50	9.92 ± 0.51	0.228
Apgar 5 min	9.99 ± 0.63	9.99 ± 0.68	9.99 ± 0.18	0.854
Apgar 1 min < 7 (n,%)			0.872	
No	8215 (99.54)	41251 (99.53)	6964 (99.56)	
Yes	24 (0.46)	193 (0.47)	31 (0.44)	
Adverse outcome				
Polyhydramnios (%)				0.530
No	47,955 (98.99)	41,033 (99.00)	6922 (98.91)	
Yes	489 (1.01)	413 (1.00)	76 (1.09)	
GHD (*n*, %)				0.001
NO	47,314 (97.67)	40,518 (97.76)	6796 (97.11)	
Yes	1130 (2.33)	928 (2.24)	202 (2.89)	
Gestational hypertension	301			
Preeclampsia	829			
Cesarean section (%)				<0.001
NO	28,018 (57.84)	24,398 (58.87)	3620 (51.73)	
Yes	20,426 (42.16)	17,048 (41.13)	3378 (48.27)	
Primary cesarean section (%)				<0.001
No	41,207 (85.06)	35,755 (86.27)	5452 (77.91)	
Yes	7237 (14.94)	5691 (13.73)	1546 (22.09)	
PD (*n*, %)				<0.001
No	46,058 (95.07)	39,520 (95.35)	6538 (93.43)	
Yes	2386 (4.93)	1926 (4.65)	460 (6.57)	
Dystocia (*n*, %)				0.001
No	46,579 (96.15)	39,800 (96.03)	6779 (96.87)	
Yes	1865 (3.85)	1646 (3.97)	219 (3.13)	
LGA (%)				<0.001
No	44,211 (91.26)	37,990 (91.66)	6221 (88.90)	
Yes	4233 (8.74)	3456 (8.34)	777 (11.10)	
Macrosomia (%)				0.004
No	46,172 (95.31)	39,550 (95.43)	6622 (94.63)	
Yes	2272 (4.69)	1896 (4.57)	376 (5.37)	
LBW (%)				<0.001
No	46,743 (96.49)	40,056 (96.65)	6687 (95.56)	
Yes	1701 (3.51)	1390 (3.35)	311 (4.44)	
ICU attendance of newborns (*n*, %)				<0.001
No	43,360 (89.51)	37,283 (89.96)	6077 (86.84)	
Yes	5084 (10.49)	4163 (10.04)	921 (13.16)	

*LBW* low birth weight, *LGA* large for gestational age, *GHD* gestational hypertensive disorder, *PD* preterm delivery before 37 weeks, Dystocia: shoulder dystocia or birth injury

^a^At delivery

^b^Categorized by WHO standard

^c^Categorized by Institute of Medicine standard

### Effects of first-trimester FPG on GDM and adverse outcomes

Table 2 presented the effects of first-trimester FPG on GDM and adverse pregnancy outcomes. The univariate analysis showed first-trimester FPG was strongly associated with risks of outcomes, including GDM, cesarean section, macrosomia, GHD, primary cesarean section, and LGA (all OR > 1, all *P* < 0.05), furthermore, the risks of GDM, primary cesarean section, and LGA was increasing with first-trimester FPG as early as it was at 4.19–4.63 mmol/L. At the same time, first-trimester FPG was a protective factor of LBW and ICU admission of the newborn (all OR < 1, all *P* < 0.05). After adjustments for multifactor, every stage of first-trimester FPG was associated with the risk of GDM (ORs for FPG 4.19–4.63, 4.63–5.11, and 5.11–7.0 mmol/L were 1.137, 1.592, and 4.031, respectively, and 95% CIs were 1.002–1.289, 1.406–1.801, and 3.513–4.625, respectively, all *P* < 0.05) and with increasing first-trimester FPG, the risks of GDM increased (the OR value increased). It was also associated with the risks of cesarean section, macrosomia, and LGA (OR for FPG 5.11–7.0 mmol/L of cesarean section: 1.128, 95% CI: 1.025–1.241; OR for FPG 5.11–7.0 mmol/L of macrosomia: 1.561, 95% CI: 1.26–1.933; OR for FPG 4.63–5.11 and 5.11–7.0 mmol/L of LGA: 1.149 and 1.426, 95% CI: 1.004–1.314 and 1.214–1.675, respectively, all *P* < 0.05) and with its increasing, the risks of LGA increased. At the same time, first-trimester FPG was a protective factor against LBW and ICU admission of the newborn (all OR < 1, all *P* < 0.05).

**Table 2:** OR for GDM and adverse pregnancy outcomes according to first-trimester FPG^a^

Outcomes	FPG	Crude ORs	*P*	Adjusted ORs	*P*
GDM	Reference	1		1	
	4.19–4.62	**1.223 (1.107~1.351)**	**<0.001**	**1.137 (1.002~1.289)**	**0.046**
	4.63–5.10	**1.825 (1.655~2.012)**	**<0.001**	**1.592 (1.406~1.801)**	**<0.001**
	5.11–6.99	**4.880 (4.378~5.439)**	**<0.001**	**4.031 (3.513~4.625)**	**<0.001**
**Adverse pregnancy outcome**					
Cesarean section	Reference	1		1	
	4.19–4.62	1.054 (0.993~1.118)	0.086	0.984 (0.916~1.057)	0.656
	4.63–5.10	**1.235 (1.163~1.312)**	**<0.001**	1.071 (0.996~1.151)	0.064
	5.11–6.99	**1.440 (1.331~1.557)**	**<0.001**	**1.128 (1.025~1.241)**	**0.014**
GHD	Reference	1		1	
	4.19–4.62	0.852 (0.700~1.036)	0.109	0.746 (0.594~0.935)	0.011
	4.63–5.10	1.021 (0.841~1.240)	0.831	0.922 (0.737~1.152)	0.475
	5.11-6.99	**1.496 (1.185~1.887)**	**0.001**	1.250 (0.956~1.634)	0.103
LGA	Reference	1		1	
	4.19–4.62	**1.251 (1.113~1.406)**	**<0.001**	1.090 (0.952~1.247)	0.213
	4.63–5.10	**1.549 (1.380~1.740)**	**<0.001**	**1.149 (1.004~1.314)**	**0.044**
	5.11–6.99	**2.124 (1.853~2.436)**	**<0.001**	**1.426 (1.214~1.675)**	**<0.001**
Polyhydramnios	Reference	1		1	
	4.19–4.62	0.841 (0.639~1.106)	0.216	0.830 (0.614~1.123)	0.228
	4.63–5.10	0.780 (0.589~1.032)	0.082	0.757 (0.555~1.033)	0.080
	5.11–6.99	0.971 (0.677~1.393)	0.874	0.971 (0.653~1.442)	0.883
Dystocia	Reference	1		1	
	4.19–4.62	0.980 (0.843~1.140)	0.793	1.081 (0.909~1.286)	0.379
	4.63–5.10	1.006 (0.864~1.171)	0.941	1.164 (0.977~1.388)	0.090
	5.11–6.99	0.946 (0.772~1.160)	0.593	1.187 (0.940~1.500)	0.150
Primary Cesarean Section	Reference	1		1	
	4.19–4.62	**1.183 (1.081~1.294)**	**<0.001**	0.893 (0.780~1.022)	0.101
	4.63–5.10	**1.532 (1.401~1.674)**	**<0.001**	0.998 (0.873~1.142)	0.982
	5.11–6.99	**1.819 (1.631~2.028)**	**<0.001**	0.977 (0.828~1.154)	0.786
ICU attendance	Reference	1		1	
	4.19-4.62	***0.879 (0.801~0.964)***	***0.006***	***0.887(0.791~0.995)***	***0.041***
	4.63–5.10	***0.890 (0.810~0.977)***	***0.014***	***0.887 (0.789~0.997)***	***0.044***
	5.11-6.99	0.943 (0.834~1.067)	0.353	0.918 (0.787~1.070)	0.274
Macrosomia	Reference	1		1	
	4.19–4.62	**1.259 (1.076~1.473)**	**0.004**	1.117 (0.932~1.338)	0.232
	4.63–5.10	**1.518 (1.299~1.773)**	**<0.001**	1.172 (0.978~1.405)	0.085
	5.11–6.99	**2.067 (1.721~2.481)**	**<0.001**	**1.561 (1.260~1.933)**	**<0.001**
LBW	Reference	1		1	
	4.19–4.62	***0.768 (0.665~0.888)***	***<0.001***	***0.771 (0.613~0.970)***	***0.026***
	4.63–5.10	***0.711 (0.613~0.824)***	***<0.001***	***0.718 (0.567~0.909)***	***0.006***
	5.11–6.99	***0.760 (0.620~0.930)***	***0.008***	0.734(0.533~1.012)	0.059
PD	Reference	1		1	
	4.19–4.62	1.021 (0.890 ~ 1.171)	0.768	1.559 (0.589~4.125)	0.371
	4.63–5.10	1.069 (0.931 ~ 1.227)	0.344	1.300 (0.486~3.482)	0.601
	5.11–6.99	1.170 (0.981 ~ 1.396)	0.081	2.232 (0.673~7.406)	0.190

Reference: First category of early FPG ≤ 4.19 mmol/L as the reference

Adjusted ORs: adjusted by maternal age, prepregancy BMI, height, delivery times, and delivery weeks

Bold: ORå 1, *P* < 0.05; Italics: OR <1, *P* < 0.05

aFPG: FPG in the first-trimester

We also conducted a subgroup analysis, which revealed both the GDM and the non-GDM subgroups had similar trends. In the GDM group, first-trimester FPG was associated with the risks of macrosomia, LGA, and dystocia (all OR > 1, all *P* < 0.05). While in the non-GDM subgroup, FPG in the first trimester was identified as a significant predictor for the risks of cesarean section, macrosomia, and LGA (all OR > 1, all *P* < 0.05), and it was a protective factor against GHD, LBW, primary cesarean section, and ICU admission of the newborn (all OR < 1, all *P* < 0.05) (Table S1, S2).

### The establishment nomogram model for predicting the risk of GDM

**Fig. 2:**
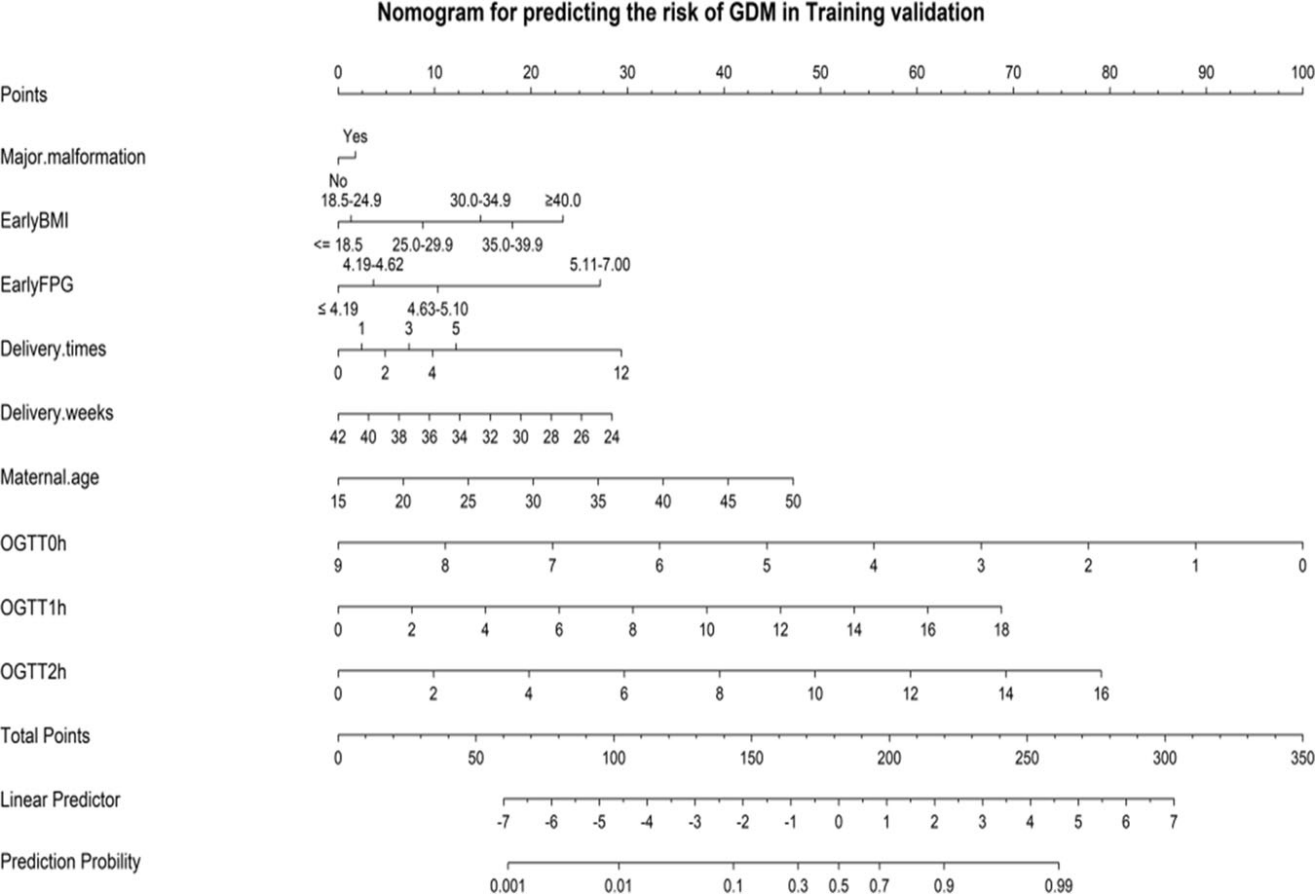
Nomogram model for predicting the risk of GDM in training validation

**Fig. 3:**
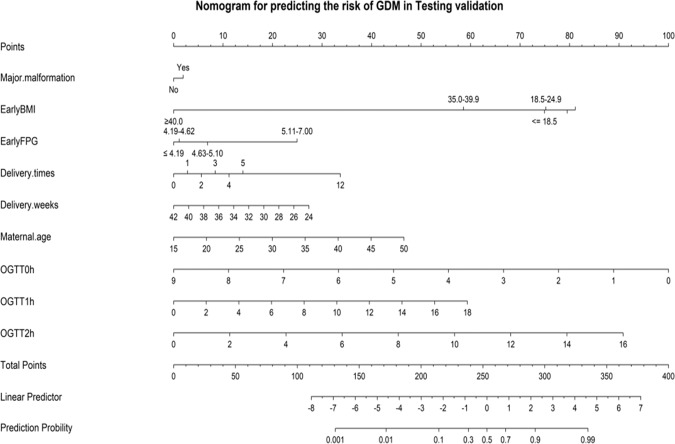
Nomogram model for predicting the risk of GDM in testing validation

**Fig. 4:**
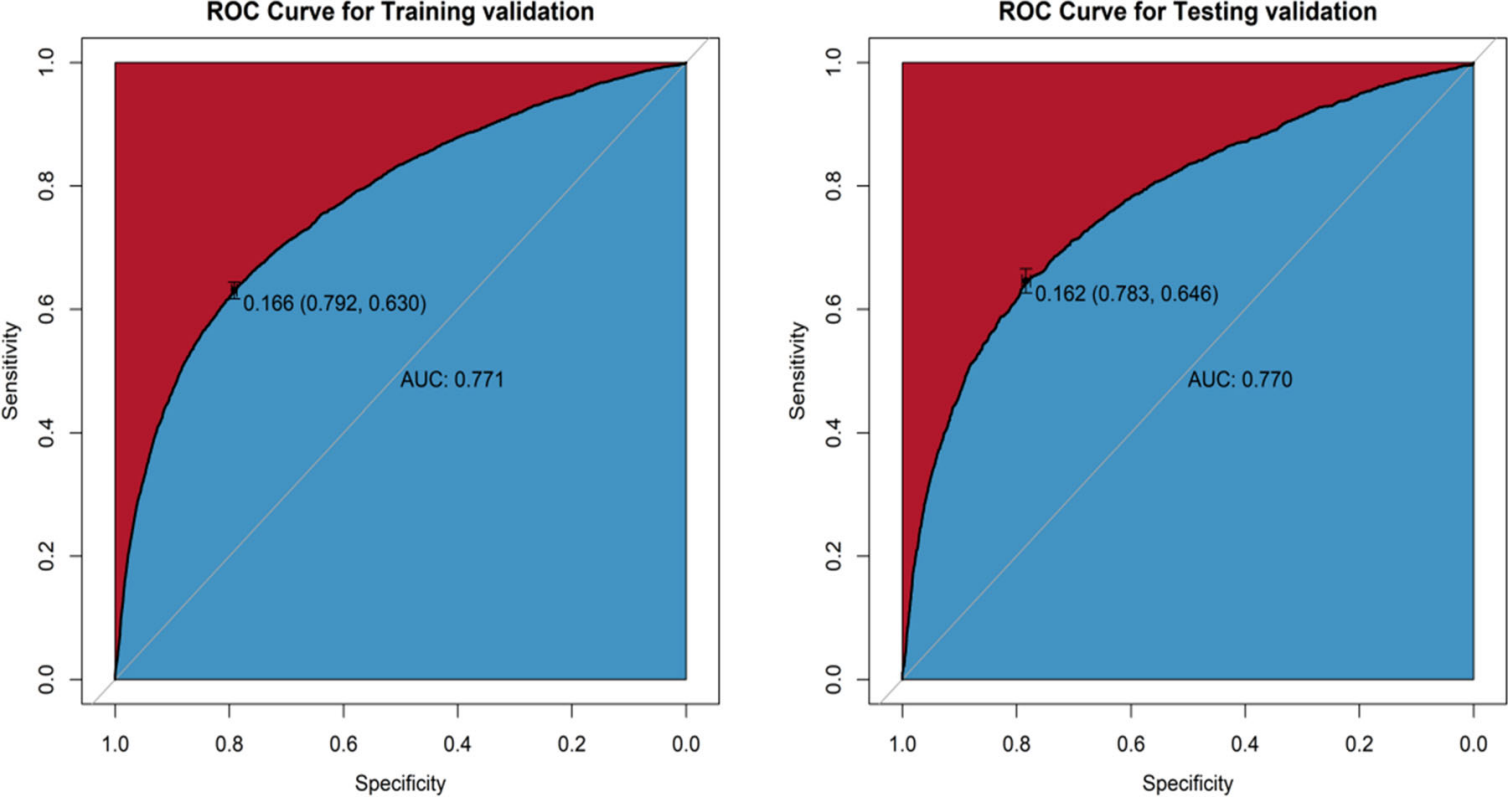
The ROC curves for predictions of the risk of GDM in the training and testing validation

**Fig. 5:**
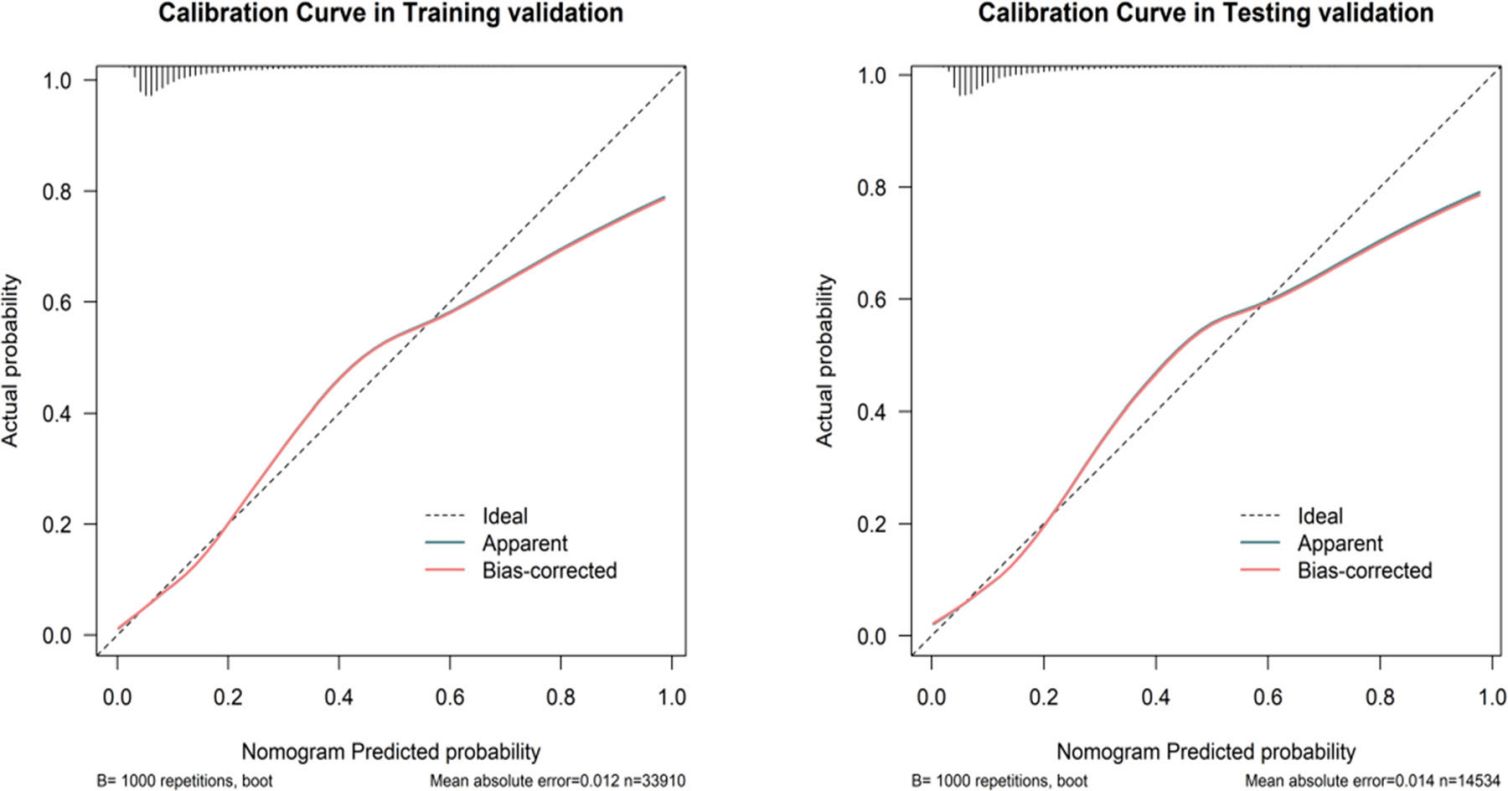
The calibration curves for predictions of the risk of GDM in the training and testing validation

Based on Table 1, maternal age, pre-pregnancy BMI, first-trimester FPG, delivery times, delivery weeks, major birth malformation of past history, GHD, OGTT at 0 h, OGTT at 1 h, and OGTT at 2 h were all significant different from pregnancies with and without GDM, a nomogram that could predict the risk of GDM was constructed. As the dataset was divided into the training and test datasets at a ratio of 7:3. The prediction results were shown in Fig. 2 and Fig. 3. As shown in Fig. 4, the training and testing validated C-indices for the nomogram were 0.771 (95%CI:0.763~0.779) and 0.770 (95%CI:0.758~0.781), respectively. Additionally, the calibration curves of the nomogram model in training and testing validation were shown in Fig. 5, from which we could see that the calibration curves of both training and testing were validation close to the ideal line, indicating an acceptable consistency between the nomogram model that predicted probability and the actual observed probability.

### Decision curve analysis used to evaluate prediction models

**Fig. 6:**
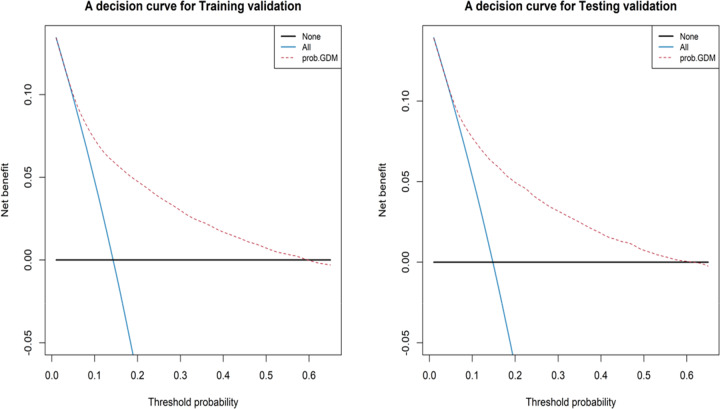
Decision curve analysis for predictions of the risk of GDM in the training and testing validation

The DCA was used to evaluate prediction models from the perspective of first-trimester FPG consequences, which revealed that compared with the conventional staging systems, the nomogram-yielded superior net clinical benefit whose threshold probabilities ranged from 0.1 to 0.6 in both the training validation and testing validation (Fig. 6). The DAC curve analysis in this clinical validity suggested that if threshold probabilities of the maternity were ranged from 0.1 to 0.6 predicted risk of GDM based on the nomogram showed more benefit than either the treat-all scheme or the treat-none.

## Discussion

This survey shows that in Shenzhen population, first-trimester FPG was not only strongly associated with GDM but also with other adverse pregnancy outcomes. In the univariate and multivariable analysis, the risks of GDM, macrosomia, primary cesarean section, and LGA can increase as early as when first-trimester FPG was at 4.19–4.63 mmol/L, and with increasing first-trimester FPG, the risks of adverse outcomes increased (the OR value increased). Furthermore, we creatively used statistical models to demonstrate that the first-trimester FPG can be used to predict GDM. The nomogram showed an acceptable consistency between the observed, validation, and nomogram-predicted probabilities, the DAC curve analysis indicated that the nomogram had important clinical application value for GDM risk prediction. The above results demonstrate that the first-trimester FPG could be used to identify adverse pregnancy risks and intervene as early as possible due to the specific metabolic changes during pregnancy.

HAPO was a prospective observational study of 25,505 pregnant women, which showed that maternal FPG is associated with increased birth weight and primary cesarean section [4, 12]. The maternal metabolic state in the first trimester may affect the outcomes of the mothers and newborns. Riskin-Mashiah et al. reported that the mild increased levels of FPG in the first trimester can lead to adverse outcomes, and they found a strong correlation between the first-trimester FPG and GDM development [5]. We also demonstrated a consistent correlation between FPG and adverse obstetric outcomes in non-GDM patients, similar to the HAPO study [4]. Our study showed that the FPG results of HAPO also applied equally to our database in Shenzhen, China. In addition, higher first-trimester FPG, though below the diagnostic FPG criterion, was associated with adverse pregnancy outcomes. In our study, as early as when first-trimester FPG was in the range of 4.19–4.63 mmol/L, the risks of GDM appeared, which may be a clue of the risks of GDM.

According to the ADA, GDM is a kind of diabetes diagnosed in the middle or late stages of pregnancy, and its symptoms are not obvious before pregnancy [8]. However, the diagnosis of GDM remains a controversial issue with multiple diagnostic criteria existing; its importance lies in its association with maternal and child health in pregnancy and later life [1]. Furthermore, it is agreed that GDM, regardless of symptoms, is associated with a significant risk of adverse perinatal outcomes. Several studies have shown that addressing GDM as early as possible can improve outcomes [1, 4], but there is much debate about its diagnosis and treatment. The main controversy involves the importance of FPG or the OGTT in the first trimester, and addressing the biases of first-trimester FPG to improve adverse outcomes for the future health of mothers and newborns stills remains discussion [1, 4]. In the study by Sacks et al., it was indicated that FPG screening for detecting early GDM was less specific, but the AUC was 0.7, which suggested that FPG still had diagnostic accuracy in predicting GDM (AUC > 0.5) [6]. Zhu et al. conducted a study of 17,186 pregnancies in China by using the IDPSG standard which showed a strong correlation between the first-trimester FPG and GDM diagnosed at 24–28 weeks of pregnancy [7]. In our research, it was also found that the diagnostic model (AUC was around 0.770) of first-trimester FPG in predicting GDM had a similar trend when using the IDPSG standard. The HAPO study indicated that there is a linear relationship between maternal FPG and macrosomia, which was similar with our study.

On the other hand, there is growing evidence showing that the first-trimester FPG is a sign of maternal and newborn health. HAPO indicated that first-trimester FPG can be used to stratify the risks and set intervention thresholds. It also showed that in the one-step OGTT, the risks of birth weight, 90th percentile of C peptides, neonatal hypoglycemia, and primary cesarean section increased linearly as the FPG of mothers increased [5, 13]. Some of our findings were accordance with the HAPO results. However, the effectiveness of FPG in predicting GDM is not generally accepted because the diagnostic criteria vary and the choice of gestational week or race is different [4, 12]. Previous studies have shown that FPG can be used to predict the risk of diabetes in later trimesters [2, 3, 13]. Riskin-Mashiah et al. studied a large number of pregnant women from Israel (*n* = 6129) and obtained similar results to ours, namely, that first-trimester FPG has an independent relationship with the risks of GDM and LGA [5].In addition, studies of lifestyle interventions to prevent GDM have shown that it works best in the early stages of pregnancy [14, 15].

The search for optimal first-trimester FPG is critical in order to avoid a comprehensive OGTT test, which needs our further investigation. Based on the IADPSG standard, Agarwal et al. pointed out that FPG had shown to be a better diagnostic tool for GDM (AUC: 0.907), and 80 mg/dL (4.44 mmol/L) is recommended as the cutoff value for FPG because of its good sensitivity (95.4%) [16]. The AUC calculated by Zhu et al. was 0.836 and the sensitivity was 87.8%. which also indicated that the first-trimester FPG of 80 mg/dL (4.44 mmol/L) is a good predictor for GDM [17]. The results by Reyes et al. showed that the cutoff value of FPG of 90 mg/dL (5.0 mmol/L) was helpful to avoid unnecessary OGTT in 0.7% of pregnant women [18]. Different studies all highlighted the usefulness and importance of using FPG alone for the diagnosis of GDM.

## Limitations

Our research has some limitations. The nutritional status of pregnancies may affect fetal growth and other perinatal outcomes, but we lack the related data. The value of PG and bilirubin of newborns is also very important for the survey, but the data are unavailable now. A number of confounded factors, such as the delivery history of microsomia, may influence the clinical decision, such as the choice of delivery method. The lack of an identified cutoff value for first-trimester FPG to date needs further exploration and indicates that the survey needs additional, more thorough research. Furthermore, first-trimester FPG is usually tested only once in the first prenatal examination, and its accuracy may be low. Our study evaluated the first-trimester FPG in a large number of pregnant women for the diagnosis of GDM, but the results may not be applicable to the general population of different races. Therefore, in future studies, it is strongly recommended to investigate the prevalence and different diagnostic methods of GDM after adjusting for all the possible associated factors in a large, multicenter population to increase the external validity of the results. Although there is no uniform optimal cutoff value for the first-trimester FPG to predict GDM, it was indicated that the diagnostic accuracy of FPG for predicting GDM was similar when using the IADPSG standard. Different studies have reported different levels of FPG for diagnosing GDM, which may be due to the study population, ethnicity, and diagnostic criteria [16, 19, 20]. The optimal cutoff value of FPG, especially in the first trimester, needs deeper and further study.

## Conclusion

Our study did find a strong correlation between first-trimester FPG and GDM, LGA, and other adverse obstetric outcomes, and identify its clinical significance in Shenzhen population, which suggested a need to reconsider the current criteria for diagnosing GDM and deal with higher first-trimester FPG. Furthermore, our study complemented the literature and suggested that first-trimester FPG may be a valuable tool for predicting GDM in southern China. Based on our study, we recommend first-trimester FPG can be used to predict GDM by the IADPSG standard, as well as a marker of obstetric risk, but the diagnosis of GDM may need comprehensive consideration. The current diagnostic standards for GDM in China should be re-examined. Further research to find the optimal cutoff value for first-trimester FPG of diagnosing GDM will be challenging.

## Supplementary information

Supplementary informationTable S1-S2

## Data Availability

The codes used during and/or analyzed during the current study are available from the corresponding authors (JM Niu) on reasonable request.
