# Supplementary material for: Fasting plasma glucose in the first trimester is related to gestational diabetes mellitus and adverse pregnancy outcomes
Source: Endocrine. 2021 Aug 3;75(1):70–81. doi: 10.1007/s12020-021-02831-w (PMC8763802; doi:10.1007/s12020-021-02831-w)
Supplement: Supplementary file 1 — Supplementary informationTable S1-S2 [file 12020_2021_2831_MOESM1_ESM.docx]

Table S1 ORs for pregnancy outcomes according to first-trimester FPG in comparison with the first category in GDM group*

| Outcomes | FPG | univariate ORs | P | Adjusted ORs | P |
| --- | --- | --- | --- | --- | --- |
| Cesarean Section | Reference | 1 |  | 1 |  |
|  | 4.19-4.62 | 1.006 (0.831 ~ 1.217) | 0.952 | 1.075 ( 0.839 ~ 1.377 ) | 0.569 |
|  | 4.63-5.10 | 1.164 (0.967 ~ 1.402) | 0.109 | 1.034 ( 0.811 ~ 1.318 ) | 0.79 |
|  | 5.11-6.99 | **1.277 (1.047 ~ 1.558)** | **0.016** | 1.102 ( 0.849 ~ 1.432 ) | 0.464 |
| Macrosomia | Reference | 1 |  | 1 |  |
|  | 4.19-4.62 | 1.605 ( 0.906 ~ 2.842 ) | 0.105 | 2.31 ( 0.98 ~ 5.445 ) | 0.056 |
|  | 4.63-5.10 | **2.132 ( 1.223 ~ 3.716 )** | **0.008** | **2.782 ( 1.201 ~ 6.444 )** | **0.017** |
|  | 5.11-6.99 | **3.143 ( 1.788 ~ 5.524 )** | **0.000** | **3.694 ( 1.58 ~ 8.638 )** | **0.003** |
| GHD | Reference | 1 |  | 1 |  |
|  | 4.19-4.62 | 0.619 ( 0.361 ~ 1.063 ) | 0.082 | 0.721 ( 0.355 ~ 1.462 ) | 0.364 |
|  | 4.63-5.10 | 0.726 ( 0.434 ~ 1.213 ) | 0.221 | 0.877 ( 0.446 ~ 1.725 ) | 0.704 |
|  | 5.11-6.99 | 1.13 ( 0.667 ~ 1.914 ) | 0.649 | 1.329 ( 0.663 ~ 2.664 ) | 0.422 |
| LBW | Reference | 1 |  | 1 |  |
|  | 4.19-4.62 | 0.733 ( 0.491 ~ 1.093 ) | 0.128 | 0.733 ( 0.373 ~ 1.439 ) | 0.367 |
|  | 4.63-5.10 | ***0.664 ( 0.449 ~ 0.983 )*** | ***0.041*** | 0.881 ( 0.458 ~ 1.694 ) | 0.703 |
|  | 5.11-6.99 | ***0.506 ( 0.323 ~ 0.792 )*** | ***0.003*** | 0.657 ( 0.313 ~ 1.381 ) | 0.268 |
| Polyhydramnios | Reference | 1 |  | 1 |  |
|  | 4.19-4.62 | 0.779 ( 0.331 ~ 1.833 ) | 0.567 | 1.189 ( 0.399 ~ 3.549 ) | 0.756 |
|  | 4.63-5.10 | 0.76 ( 0.33 ~ 1.75 ) | 0.520 | 1.007 ( 0.339 ~ 2.987 ) | 0.99 |
|  | 5.11-6.99 | 0.983 ( 0.411 ~ 2.352 ) | 0.969 | 1.637 ( 0.537 ~ 4.988 ) | 0.386 |
| PD | Reference | 1 |  | 1 |  |
|  | 4.19-4.62 | 0.898 ( 0.622 ~ 1.297 ) | 0.566 | 2.205 ( 0.08 ~ 60.427 ) | 0.64 |
|  | 4.63-5.10 | 0.888 ( 0.62 ~ 1.271 ) | 0.515 | 1.036 ( 0.038 ~ 28.095 ) | 0.983 |
|  | 5.11-6.99 | 0.816 ( 0.552 ~ 1.205 ) | 0.306 | 1.239 ( 0.034 ~ 45.524 ) | 0.907 |
| Primary Cesarean Section | Reference | 1 |  | 1 |  |
|  | 4.19-4.62 | 1.179 ( 0.916 ~ 1.516 ) | 0.200 | 1.265 ( 0.851 ~ 1.881 ) | 0.245 |
|  | 4.63-5.10 | **1.557 ( 1.221 ~ 1.987 )** | **0.000** | 1.19 ( 0.806 ~ 1.757 ) | 0.381 |
|  | 5.11-6.99 | **1.682 ( 1.302 ~ 2.173 )** | **0.000** | 1.273 ( 0.845 ~ 1.917 ) | 0.249 |
| LGA | Reference | 1 |  | 1 |  |
|  | 4.19-4.62 | 1.208 ( 0.84 ~ 1.737 ) | 0.308 | 1.419 ( 0.864 ~ 2.33 ) | 0.167 |
|  | 4.63-5.10 | **1.72 ( 1.213 ~ 2.44 )** | **0.002** | **1.75 ( 1.081 ~ 2.833 )** | **0.023** |
|  | 5.11-6.99 | **2.302 ( 1.607 ~ 3.298 )** | **0.000** | **2.28 ( 1.393 ~ 3.731 )** | **0.001** |
| ICU attendance of  newborns | Reference | 1 |  | 1 |  |
|  | 4.19-4.62 | 0.981 ( 0.746 ~ 1.292 ) | 0.893 | 1.032 ( 0.712 ~ 1.495 ) | 0.868 |
|  | 4.63-5.10 | 0.882 ( 0.674 ~ 1.156 ) | 0.363 | 0.933 ( 0.647 ~ 1.344 ) | 0.708 |
|  | 5.11-6.99 | 0.937 ( 0.702 ~ 1.25 ) | 0.656 | 0.985 ( 0.666 ~ 1.458 ) | 0.94 |
| Dystocia | Reference | 1 |  | 1 |  |
|  | 4.19-4.62 | 1.486 ( 0.8 ~ 2.761 ) | 0.210 | 1.706 ( 0.758 ~ 3.838 ) | 0.197 |
|  | 4.63-5.10 | 1.329 ( 0.72 ~ 2.452 ) | 0.363 | 2.072 ( 0.937 ~ 4.58 ) | 0.072 |
|  | 5.11-6.99 | 1.591 ( 0.842 ~ 3.004 ) | 0.152 | **2.388 ( 1.046 ~ 5.451 )** | **0.039** |

FPG: FPG in the first-trimester

Reference: First category of early FPG≤4.19 mmol/L as the reference

Adjusted ORs:adjusted by maternal age,prepregancy BMI,height,delivery times and delivery weeks

Table S2 ORs for pregnancy outcomes according to first-trimester FPG in comparison with the first category in non-GDM group*

| Outcomes | FPG | univariate ORs | P | Adjusted ORs | P |
| --- | --- | --- | --- | --- | --- |
| Cesarean Section | Reference | 1 |  | 1 |  |
|  | 4.19-4.62 | **1.054 (0.989 ~ 1.122 )** | **0.104** | 0.975 ( 0.905 ~ 1.051 ) | 0.51 |
|  | 4.63-5.10 | **1.222 ( 1.147 ~ 1.303 )** | **0.000** | 1.078 ( 0.999 ~ 1.163 ) | 0.053 |
|  | 5.11-6.99 | **1.378 ( 1.259 ~ 1.508 )** | **0.000** | **1.142 ( 1.026 ~ 1.271 )** | **0.015** |
| Macrosomia | Reference | 1 |  | 1 |  |
|  | 4.19-4.62 | **1.234 ( 1.048 ~ 1.453 )** | **0.012** | 1.069 ( 0.888 ~ 1.288 ) | 0.48 |
|  | 4.63-5.10 | **1.462 ( 1.242 ~ 1.722 )** | **0.000** | 1.096 ( 0.909 ~ 1.322 ) | 0.336 |
|  | 5.11-6.99 | **1.86 ( 1.512 ~ 2.288 )** | **0.000** | **1.368 ( 1.079 ~ 1.734 )** | **0.01** |
| GHD | Reference | 1 |  | 1 |  |
|  | 4.19-4.62 | 0.886 ( 0.718 ~ 1.094 ) | 0.262 | 0.749 ( 0.589 ~ 0.952 ) | 0.018 |
|  | 4.63-5.10 | 1.06 ( 0.86 ~ 1.307 ) | 0.584 | 0.932 ( 0.735 ~ 1.181 ) | 0.56 |
|  | 5.11-6.99 | **1.456 ( 1.109 ~ 1.91 )** | **0.007** | 1.255 ( 0.927 ~ 1.7 ) | 0.142 |
| LBW | Reference | 1 |  | 1 |  |
|  | 4.19-4.62 | ***0.767 ( 0.656 ~ 0.896 )*** | ***0.001*** | ***0.776 ( 0.608 ~ 0.99 )*** | ***0.041*** |
|  | 4.63-5.10 | ***0.696 ( 0.592 ~ 0.818 )*** | ***0.000*** | ***0.685 ( 0.531 ~ 0.883 )*** | ***0.004*** |
|  | 5.11-6.99 | 0.808 ( 0.639 ~ 1.022 ) | 0.075 | 0.761 ( 0.526 ~ 1.101 ) | 0.147 |
| Polyhydramnios | Reference | 1 |  | 1 |  |
|  | 4.19-4.62 | 0.847 ( 0.635 ~ 1.132 ) | 0.262 | 0.802 ( 0.585 ~ 1.099 ) | 0.171 |
|  | 4.63-5.10 | 0.777 ( 0.576 ~ 1.047 ) | 0.098 | 0.738 ( 0.533 ~ 1.023 ) | 0.068 |
|  | 5.11-6.99 | 0.925 ( 0.606 ~ 1.413 ) | 0.719 | 0.868 ( 0.549 ~ 1.373 ) | 0.545 |
| PD | Reference | 1 |  | 1 |  |
|  | 4.19-4.62 | 1.031 ( 0.889 ~ 1.196 ) | 0.686 | 1.518 ( 0.546 ~ 4.223 ) | 0.424 |
|  | 4.63-5.10 | 1.068 ( 0.919 ~ 1.241 ) | 0.390 | 1.356 ( 0.481 ~ 3.826 ) | 0.564 |
|  | 5.11-6.99 | 1.173 ( 0.953 ~ 1.442 ) | 0.132 | 2.836 ( 0.783 ~ 10.272 ) | 0.112 |
| Primary Cesarean Section | Reference | 1 |  | 1 |  |
|  | 4.19-4.62 | **1.17 ( 1.063 ~ 1.289 )** | **0.001** | 0.842 ( 0.728 ~ 0.972 ) | 0.019 |
|  | 4.63-5.10 | **1.464 ( 1.33 ~ 1.612 )** | **0.000** | 0.966 ( 0.837 ~ 1.116 ) | 0.638 |
|  | 5.11-6.99 | **1.567 ( 1.378 ~ 1.783 )** | **0.000** | 0.907 ( 0.749 ~ 1.098 ) | 0.317 |
| LGA | Reference | 1 |  | 1 |  |
|  | 4.19-4.62 | **1.253 ( 1.108 ~ 1.418 )** | **0.000** | 1.067 ( 0.927 ~ 1.228 ) | 0.368 |
|  | 4.63-5.10 | **1.499 ( 1.325 ~ 1.696 )** | **0.000** | 1.094 ( 0.95 ~ 1.261 ) | 0.212 |
|  | 5.11-6.99 | **1.926 ( 1.646 ~ 2.254 )** | **0.000** | **1.276 ( 1.064 ~ 1.53 )** | **0.009** |
| ICU attendance of  newborns | Reference | 1 |  | 1 |  |
|  | 4.19-4.62 | ***0.859 ( 0.779 ~ 0.948 )*** | ***0.003*** | ***0.871 ( 0.772 ~ 0.983 )*** | ***0.025*** |
|  | 4.63-5.10 | ***0.874 ( 0.79 ~ 0.965 )*** | ***0.008*** | ***0.88 ( 0.777 ~ 0.995 )*** | ***0.042*** |
|  | 5.11-6.99 | ***0.86 ( 0.743 ~ 0.996 )*** | ***0.044*** | 0.881 ( 0.738 ~ 1.051 ) | 0.159 |
| Dystocia | Reference | 1 |  | 1 |  |
|  | 4.19-4.62 | 0.955 ( 0.817 ~ 1.116 ) | 0.561 | 1.055 ( 0.883 ~ 1.26 ) | 0.558 |
|  | 4.63-5.10 | 1.008 ( 0.861 ~ 1.18 ) | 0.924 | 1.123 ( 0.936 ~ 1.346 ) | 0.211 |
|  | 5.11-6.99 | 0.926 ( 0.735 ~ 1.167 ) | 0.514 | 1.087 ( 0.838 ~ 1.41 ) | 0.53 |

FPG:FPG in the first-trimester

Reference: First category of early FPG≤4.19 mmol/L as the reference

Adjusted ORs:adjusted by maternal age,prepregancy BMI,height,delivery times and delivery weeks
